# Statement of Retraction: Long noncoding RNA MALAT1 knockdown inhibits progression of anaplastic thyroid carcinoma by regulating miR-200a-3p/FOXA1

**DOI:** 10.1080/15384047.2026.2746185

**Published:** 2026-10-08

We, the Editors and Publisher of *Cancer Biology & Therapy*, have retracted the following article:

Gou, L., Zou, H., & Li, B. (2019). Long noncoding RNA MALAT1 knockdown inhibits progression of anaplastic thyroid carcinoma by regulating miR-200a-3p/FOXA1. *Cancer Biology & Therapy*, *20*(11), 1355–1365. https://doi.org/10.1080/15384047.2019.1617567

Following publication, concerns were raised in 2026 regarding the image integrity of Western blot figures and RT-qPCR methodology and results described in the article. When approached for an explanation, the authors did not respond to our queries.

As verifying the validity of published work is core to the integrity of the scholarly record, we are therefore retracting the article. The corresponding author has been informed.

We have been informed in our decision-making by our editorial policies and the COPE guidelines.

The retracted article will remain online to maintain the scholarly record, but it will be digitally watermarked on each page as ‘Retracted’.

